# Editorial: Continuous flowering: A much- needed ornamental revolution for floricultural crops

**DOI:** 10.3389/fpls.2023.1172215

**Published:** 2023-03-13

**Authors:** Sagheer Ahmad, Fengxi Yang, Tangchun Zheng, Toshi Marie Foster

**Affiliations:** ^1^ Institute of Environmental Horticulture, Guangdong Academy of Agricultural Sciences, Guangzhou, China; ^2^ Guangdong Laboratory of Lingnan Modern Agriculture, Guangzhou, China; ^3^ Beijing Key Laboratory of Ornamental Plants Germplasm Innovation and Molecular Breeding, National Engineering Research Center for Floriculture, Beijing Forestry University, Beijing, China; ^4^ Beijing Laboratory of Urban and Rural Ecological Environment, Engineering Research Center of Landscape Environment of Ministry of Education, Beijing Forestry University, Beijing, China; ^5^ Key Laboratory of Genetics and Breeding in Forest Trees and Ornamental Plants of Ministry of Education, School of Landscape Architecture, Beijing Forestry University, Beijing, China; ^6^ The New Zealand Institute for Plant & Food Research Limited, Palmerston North Research Centre, Palmerston North, New Zealand

**Keywords:** recurrent flowering, genetic regulation, regulatory pathways, transcriptome studies, biotechnology, floriculture crops

Floricultural crops are an integral part of our routine life, with uses ranging from aesthetics to food and medicine. The floriculture industry contributes significantly to the global economy owing to the extensive use of flowers and their products for occasional and routine uses. Therefore, a large proportion of the scientific community is engaged in research on the innovation of floriculture crops as a means to meet the rapidly growing demand for flowers in different aspects of life. Seasonal flowering is the established pattern of floricultural crops, wherein flowering occurs at a specific season each year. Some of the most beautiful flowers can only be seen within a short-lived time frame. Worthy of note are the orchids, which are the most demanded flowers in the world that usually produce flowering after a long time (2-3 years). Crops with the ability to flower continuously throughout the year make a clear difference in their market success and preference in the floriculture industry. Studies on the genetic basis of continuous flowering patterns and their subsequent application to seasonal flowering crops can offer the best program for revolutionizing the continuous availability of the most precious floriculture crops. To explore this interesting research direction, this editorial showcases recent findings and novel insights into “Continuous Flowering” to broaden our current understanding of the flowering time pattern of ornamental plants.

## Flowering regulation in chrysanthemum and osmanthus

Chrysanthemum is one of the most important cash crops in the floriculture industry. Therefore, its flowering time regulation is a hot topic. Liu et al. presented a whole-transcriptome study to screen differentially expressed genes (DEGs) between ray and disc florets to identify flowering regulatory genes in *Chrysanthemum morifolium*. A total of 8,359 DEGs were found between the disc and ray florets. Of these, 3,005 were upregulated and 5,354 were downregulated in the disc florets. *TM6* (Class B floral homeotic MADS-box TF) was the most important gene expressed in the disc florets. The ectopic expression of *CmTM6-mu* in *Arabidopsis thaliana* shortened the flowering time in the transgenic plants.

In another study, Li et al. showed that continuous flowering is caused by compound expression of FTLs in *Chrysanthemum morifolium* × *Leucanthemum paludosum* intergeneric hybridization. The ectopic expression of *Leucanthemum paludosumTFLs* (*LpTFLs*) in *A. thaliana* caused early flowering, suggesting that *LpFTLs* affect flowering time. Compound expression of *FTLs* in *C. morifolium* × *L. paludosum* intergeneric hybridization causes serious heterosis in the hybrid offspring. Moreover, continuous flowering seems to be accompanied by hybrid weakness under the balance of vegetative and reproductive growth.

Sweet osmanthus (*Osmanthus fragrans* Lour.), exhibits two types of flowering characteristics: once-flowering (OF) habit and continuous flowering (CF) habit. Wang et al. analyzed the flowering phenology shifts of OF and CF habits in sweet osmanthus through histology, microscopy, and transcriptional assays. Transcriptional activity analysis of flowering-related genes identified three floral integrators, *OfFT*, *OfTFL1*, and *OfBFT*, with a differential expression during the floral transition process in OF and CF habits. A function evaluation suggested *OfFT* as a flowering activator and *OfBFT* as a flowering inhibitor. Moreover, a yeast one-hybrid assay indicated *OfSPL8* as a common upstream transcription factor of *OfFT* and *OfBFT*, signifying the significant role of *OfSPL8* in the regulation of continuous flowering.

## Flowering regulation in woody bamboo and *Prunus*

Woody bamboo has peculiar flowering characteristics, with intervals spanning from several years to more than 100 years. Elucidating flowering time regulation in bamboo could be beneficial for both humans and wildlife. To explore the mechanism of flowering time regulation in *Bambusa oldhamii* ‘Xia Zao’ ZSX, a transcriptome sequencing was performed by Zhao et al. to illustrate the genes involved in flower development. Seventeen differentially expressed orthogroups associated with flowering were identified between non-flowering and flowering culm buds. Six regulators were found in the photoperiod pathway, which were confirmed by mapping the flowering time network in rice. The key regulators, including *Rice FT-like 1* (*RFT1*) and *Heading date* (*Hd3a*), were found to integrate upstream signaling into the downstream effectors, suggesting the presence of an intact photoperiod pathway that switches flowering on/off in *B. oldhamii* ‘Xia Zao’.

Another study by Zhao et al. emphasized the integral roles of *Prunus mume DORMANCY-ASSOCIATED MADS-BOX* genes (*PmDAMs*) and *SHORT VEGETATIVE PHASE* genes (*PmSVPs*) in the flower organ development and dormancy cycle. They proposed that PmSVP1 and PmSVP2 could combine with PmDAM1 to affect flower organogenesis and interact with PmDAM5 and PmDAM6 to control flower bud dormancy.

## Role of TCP gene family in flowering regulation

TCP proteins (TCPs) are plant-specific transcription factors (TFs), which are named after *TEOSINTE BRANCHED1* (*TB1*) in *Zea mays*, *CYCLOIDEA* (*CYC*) in *Antirrhinum majus*, and *PROLIFERATING CELL FACTORS 1* and *2* (*PCF1* and *PCF2*) in *Oryza sativa*. TCP proteins are involved in a broad range of physiological processes of plant growth and development. However, their origin and evolutionary history are not fully revealed. Considering that TCPs play indispensable roles in flowering regulation, Wang et al. presented a genome-wide survey of *TCP* genes in 59 species (including 42 genomes and 17 transcriptomes), covering all the main lineages of green plants, and rebuilt the evolutionary history of this family. They suggested that the origin of *TCP* genes predated the emergence of land plants, possibly in the common ancestor of Phragmoplastophyta.

## Light quality impacts flower quality

Light quality has a strong influence on the growth and flower quality of ornamental plants. The *Hippeastrum* genus is famous for its large and colorful flowers of great ornamental value. Plenty of research has been invested to manipulate its flowering time. However, the optimum light quality for the growth and flowering of *Hippeastrum* remains to be validated. Wang et al. inspected the effect of the red/blue light ratio of light-emitting diodes (LEDs) on the growth and flowering quality of *H. hybrid* ‘Red Lion’. The optimal intensity of red and blue light promoted carbohydrate accumulation and early flowering and elongated the flowering period of *H. hybrid*.

Flowering regulation is a complex process that requires the strict management of genetic and environmental cues. Manipulation of flowering habits, thus, necessitates long-term functional studies at genetic levels. The studies mentioned above can be a foreground to set up the next targets for altered ornamental plants with desired flowering characteristics.

## Author contributions

SA drafted the manuscript. All the authors contributed to the article and approved the submitted version.

